# Effect of electromagnetic fields on human osteoarthritic and non-osteoarthritic chondrocytes

**DOI:** 10.1186/s12906-017-1868-z

**Published:** 2017-08-14

**Authors:** Julia Isabelle Redeker, Bärbel Schmitt, Nele Pascale Grigull, Christian Braun, Andreas Büttner, Volkmar Jansson, Susanne Mayer-Wagner

**Affiliations:** 1 Department of Orthopaedic Surgery, Physical Medicine and Rehabilitation, Ludwig-Maximilians-University, Munich, Germany; 2 Institute for Legal Medicine, Ludwig-Maximilians-University, Munich, Germany; 3 Institute of Forensic Medicine, Rostock University Medical Center, Rostock, Germany

**Keywords:** Electromagnetic field, Human chondrocytes, Osteoarthritis, Real-time PCR, Cartilage specific genes

## Abstract

**Background:**

Studies of the effects of electromagnetic fields (EMFs) on cartilaginous cells show a broad range of outcomes. However EMFs are not yet clinically applied as standard treatment of osteoarthritis, as EMF effects are showing varying outcomes in the literature. The aim of this study was to examine effects of EMFs (5 mT or 8 mT) on osteoarthritic (OA) and non-OA chondrocytes in order to investigate whether EMF effects are related to chondrocyte and EMF quality.

**Methods:**

Pellets of human OA and non-OA chondrocytes were exposed to a sinusoidal 15 Hz EMF produced by a solenoid. Control groups were cultivated without EMF under standard conditions for 7 days. Cultures were examined by staining, immunohistochemistry and quantitative real-time PCR for RNA corresponding to cartilage specific proteins (COL2A1, ACAN, SOX9).

**Results:**

OA chondrocytes increased the expression of COL2A1 and ACAN under 5 mT EMF compared to control. In contrast no changes in gene expression were observed in non-OA chondrocytes. OA and non-OA chondrocytes showed no significant changes in gene expression under 8 mT EMF.

**Conclusion:**

A 5 mT EMF increased the expression of cartilage specific genes in OA chondrocytes whereas in non-OA chondrocytes no changes in gene expression were observed. An 8 mT EMF however showed no effect altogether. This suggests that EMF effects are related to EMF but also to chondrocyte quality. Further studies about the clinical relevance of this effect are necessary.

## Background

Osteoarthritis (OA) refers to a syndrome of joint pain accompanied by functional limitation and reduced quality of life. OA has a high prevalence, which is expected to increase due to the aging population and currently affects over 40 million Europeans [1]. It is one of the leading causes of pain and disability in everyday life worldwide. The severity of knee and hip OA disability has been associated with a significant increase in all-cause mortality and serious cardiovascular disease events due to the avoidance of physical activities that exacerbate symptoms [2]_._ The socioeconomic impact is very high due to the enormous and dramatically escalating burden of this disease [3]. As there is no cure for OA, a multimodal pharmacologic and non-pharmacologic approach followed by joint replacement is recommended by most guidelines. However, non-invasive multimodal approaches often only result in short term improvement or fail altogether. Additional strategies are needed to treat pain and improve joint function while minimizing side effects. Biological effects of electromagnetic fields (EMFs) in tissues were first described by Fukada and Yasuda [4], who investigated the piezoelectric phenomenon in bone. Electric and electromagnetic potentials were found to not only affect osteogenesis but also have chondroprotective properties [5]. In animal studies EMFs reduced knee OA lesions [6] and have the ability to antagonize the catabolic activity of cytokines in cartilage explants [5, 7, 8]. Other studies indicate that EMFs may affect intracellular calcium concentration triggering proteoglycan synthesis [9, 10]. EMF therapy has been shown to partially reduce pain and improve mobility for the treatment of OA as compared to placebo [2, 4, 11]. However, the question whether EMFs should be used as standard therapy for the treatment of OA remains open.

Experimental studies on EMFs are inconsistent with regard to electromagnetic fields, effector cells or tissues and effector responses. Two current reviews follow the same search approach and analyzed the effect of EMF for the treatment of OA as compared to placebo [2, 4]. The question remains whether EMF is generally a form of therapy suitable for everybody, or whether there are specific patients who might profit from a specific form of EMF therapy. Previous studies have shown that EMFs increase the chondrogenic potential of human mesenchymal stem cells (hMSCs) during chondrogenic differentiation [12]. Most astonishing was the fact that positive EMF effects occurred here only under suboptimal and not under optimal cellular conditions [12]. It is not known whether the suboptimal chondrocytes seen in OA respond differently to EMF than healthy chondrocytes. The aim of this study was to compare the effects of EMFs on OA versus non-OA chondrocytes. To our knowledge this is the first approach to specifically address this question. We further investigated whether the effect could be indeed by any EMF or whether a specific EMF is necessary. We hypothesized that specific EMFs would show more effects on degenerated osteoarthritic than on non-osteoarthritic chondrocytes.

## Methods

### Cell culture

**Fig. 1:**
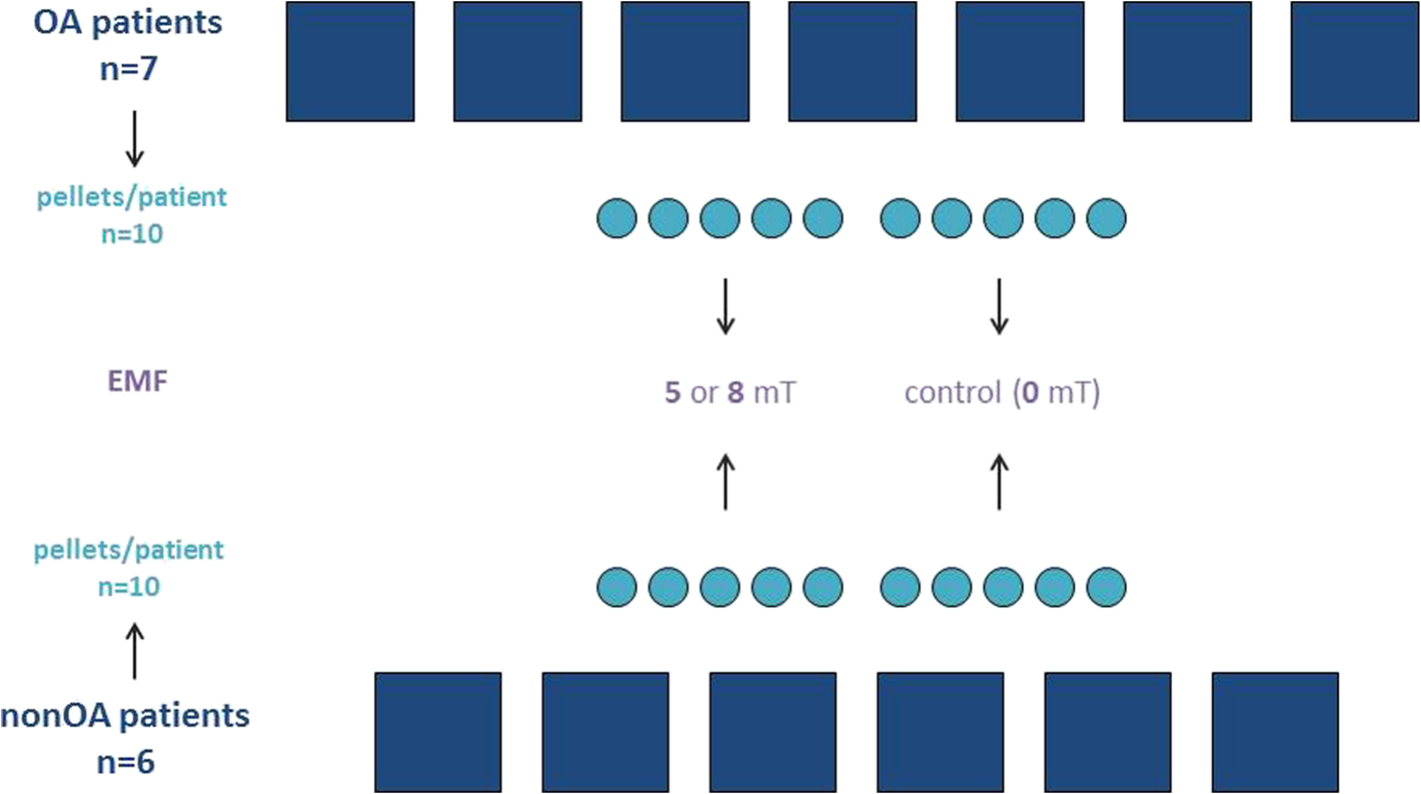
Chart of the experiment with 7 OA patients and 6 nonOA patients. During the experiment 5 pellet cultures per patient were exposed to an EMF (5 mT or 8 mT). As control group 5 pellet cultures from the same patient were cultivated in a second incubator without a solenoid for 7 days

Human OA articular cartilage was obtained from adult patients (patient 1-7) during total knee replacement (*n* = 7) (1 male, 6 female; mean age 67; range 58 - 85). Non-OA articular cartilage was obtained from young patients (patient 8-13) during triple arthrodesis performed as salvage procedure for refactory clubfoot (*n* = 3) or from human knee condyles *of deceased patients after trauma devoid of involvement* of the knee harvested within 12 h of death (*n* = 3) (together 5 male, 1 female; mean age 15; range 13 - 20) (Fig. 1). The study was approved by the Ludwig-Maximilians-University medical center ethics committee. Chondrocytes were isolated by enzymatic treatment using pronase (Roche Diagnostics, Mannheim, Germany) and collagenase (Sigma-Aldrich, St. Louis, MO) and cultured at 37 °C in a humidified atmosphere in Dulbecco’s modified Eagle’s medium (DMEM)/Hams F-12 (1:1; Biochrom, Berlin, Germany) containing 10% fetal calf serum (PAA, Pasching, Austria), 1% MEM–amino acids (Biochrom, Berlin, Germany), 25 μg/ml ascorbic acid (Sigma-Aldrich, St. Louis, MO), 50 IU/ml penicillin–streptomycin and 0.25 μg/ml Amphotericin B (Biochrom, Berlin, Germany). The medium was changed every second day. At 100% confluence, cells were passaged using 0.05% Trypsin containing 0.02% EDTA (Biochrom, Berlin, Germany) and were expanded in monolayer triple flasks (Nunc, Rosklide, Denmark). Experiments were performed with chondrocytes at passage 2. At day zero, 4 × 10^5^ chondrocytes were lysed in 1 ml TRIzol Reagent (Life Technologies, Carlsbad, CA) and kept at −80 °C for further RNA-analysis. To form pellet cultures (*n* = 10 for each patient), 4 × 10^5^ cells were centrifuged at 150 *g* for 5 min in a 15 ml polypropylene tube (TPP, Trasadingen, Switzerland). All pellet cultures were cultured for 48 h under the same conditions as mentioned above to allow pellet formation. Experiments were started by transferring pellets into 1.5 ml reaction tubes (Eppendorf, Hamburg, Germany), containing 1 ml of medium and sealed by breathe easy sealing membranes (Sigma-Aldrich, Steinheim, Germany). During the experiment 5 pellet cultures per patient (3 pellets for RNA-analyses and 2 pellets for histology and immunohistochemistry (IHC)) were exposed to EMF (5 mT or 8 mT) respectively within a solenoid. The respective control pellet cultures (*n* = 5) from the same patient were cultivated in a second incubator without a solenoid for 7 days, established as control [12] (Fig. 1). All experiments were performed for 7 days, with media being changed every second day.

### Low frequency sinusoidal EMF

EMFs were generated with a solenoid (FA-P6-K, Neue Magnetodyn, Munich, Germany) combined with a frequency generator (M80, Neue Magnetodyn, Munich, Germany) as described by Mayer-Wagner et al. [12]. For experiments, pellets were cultured in 1.5 ml Eppendorf-Safe-Lock tube (Eppendorf, Hamburg, Germany) fixed in a tube-rack inside the coil. To allow gas-exchange tubes were closed with a permeable sealing membrane (Sigma-Aldrich, Steinheim, Germany). The EMF waveform was measured with a teslameter (Bell 640, F.W. Bell, Orlando, FL) inside the coil at the position of the pellet cultures.

In this configuration, the magnetic field is in parallel to the long axis of the base of the tube-rack. The electric field induced by the magnetic field in the plane of the pellets can be calculated with good approximation by the following equation: *E*_*ymax*_ *= 2h*πf*B*_*x*_ *πf* [13], where *B* is the peak value of the magnetic flux density, fis its frequency and *h* is the height of the pellets. With the given values the maximum induced electric field is below 2 mV/m.

The particular waveform, amplitude and sinusoidal frequency of 15 Hz have been established previously in vivo [14, 15] and in vitro [12, 16, 17]. The homogeneity of the magnetic field within the solenoid was assured by measuring the spatial distribution of the magnetic flux density with a teslameter. The geomagnetic field in the incubator was determined to be 45 μT parallel to the rotation axis of the solenoid. The field (either 5 mT or 8 mT) was applied every 8 h for 45 min during the course of the 7 day experiment. The waveform was a pure sinusoidal wave with a total harmonic distortion <1%. The 8 mT field was applied in order to use an unspecific field, which has so far not been shown in the literature to induce effects but is still close to the 5 mT field described to produce an effect.

### Histology

Pellet cultures (2 with EMF treatment and 2 without treatment for each patient) were washed in phosphate buffered saline (PBS) pH 7.4, incubated in 5% sucrose in PBS (21 °C, 15 min), dry embedded in Tissue-Tek (Sakura, Zoeterwoude, Netherlands) and frozen at −20 °C. Serial cryosections (8 μm) were prepared by mounting pellet cultures on SuperFrost glass slides (Menzel-Gläser, Braunschweig, Germany). Cryosections were fixed in acetone (AppliChem, Darmstadt, Germany) and dried at room temperature (RT). Serial sections were stained in triplicate with 0.75% safranin-O (Fluka, Buchs, Switzerland) and 0.02% fast green (Chroma, Münster, Germany).

### Immunohistochemistry

**Fig. 2:**
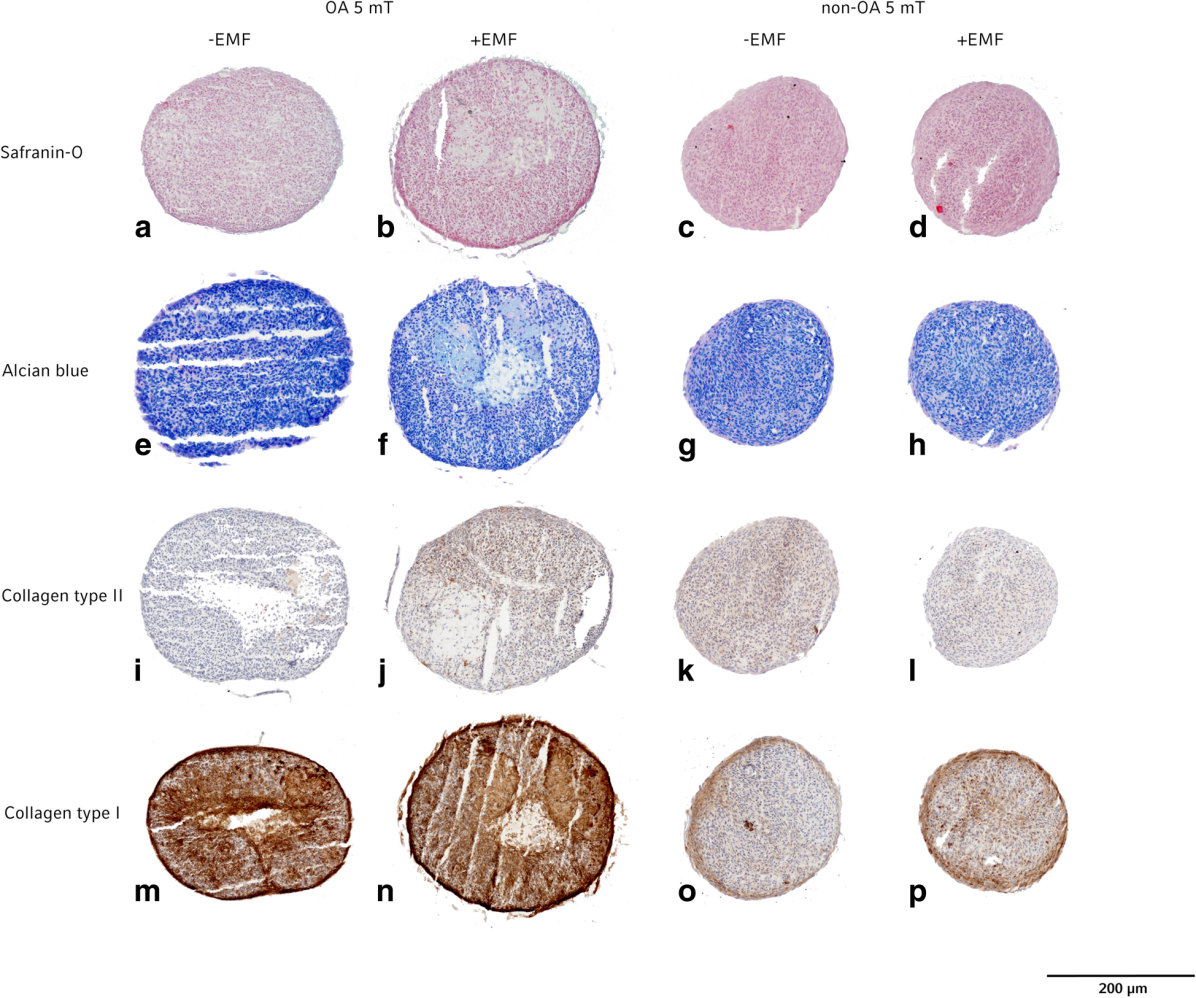
Histology and Immunohistochemistry (IHC) staining of one OA-patient and one non-OApatient pellet cultures after 5 mT EMF exposition (**b, f, j, n, d, h, l, p**) or without EMF exposition **(a, e, i, m, c, g, k, o**). Safranin-O staining (**a, b, c, d**) and alcian blue staining (**e, f, g, h**) was positive for all pellet cultures. IHC for collagen type II was much more obvious for OA pellet culture after EMF exposition (**j**) and non-OA pellet cultures with and without EMF exposition (**k, l**). IHC for collagen type I of OA pellet cultures (**m, n**) showed a weaker staining than non-OA pellet cultures (**o, p**)

Pellet sections were fixed in acetone (AppliChem, Darmstadt, Germany) (10 min, RT) and washed in washing buffer (PBS with 0.2% 5 Brij L23 Solution (Sigma-Aldrich, Steinheim, Germany)). After blocking endogenous tissue peroxidase activity with 0.3% H_2_O_2_ (Merck, Darmstadt, Germany) in aqua dest (4 min, RT) pellet sections were treated with 0.25 U/ml chondroitinase AC from *Flavobacterium Heparium* (Sigma-Aldrich, Steinheim, Germany) in PBS (30 min, 37 °C) followed by washing in washing buffer. Afterwards pellet sections were incubated with monoclonal mouse antibodies (mABs) against collagen type I (Sigma-Aldrich, Saint Louis, MO) diluted in 1:2000 in mAB-dilution-soluion (DCS, Hamburg, Germany) or collagen type II (DSHB, University of Iowa, IA) 1:6 in mAB-dilution-solution (30 min, RT). For negative controls the mABs were omitted. Pellet sections were washed in washing buffer and then incubated with a biotinylated horse-anti-mouse IgG (Vector Laboratories, Burlingame, CA) diluted 1:200 in mAB-dilution-solution (30 min, RT). Afterwards pellet sections were incubated with avidin-biotin-peroxidase-complex (ABC) (Vector Laboratories, Burlingame, CA). After repeated washing, the bound mABs were visualized with 3.3 diamino benzidine tetrahydrochlorhide (DAB) (Vector Laboratories, Burlingame, CA) for approximately 4 min without light. The reaction was stopped with aqua dest. And the sections were counterstained with haematoxylin (AppliChem, Maryland, CA) and embedded in Eukitt (O-Kindler, Freiburg, Germany). Representative images were obtained using a PreciPoint M8 microscope (PreciPoint, Freising, Germany) (Fig. 2).

### Visual histological grading system

The morphology of pellet cultures was graded according to a visual histological grading system for generated neocartilage [18]. Two independent and blinded observers evaluated each pellet in 3 categories described below and assigned scores ranging from 0 to 3. Categories were added with equal weight to give a total score (Table 1).

**Table 1:** Visual histological grading system

	A	B	C	total
OA				
EMF 0 mT	1	1	1	3
EMF 5 mT	2	2	1	5
EMF 8 mT	1	1	1	3
Non-OA				
EMF 0 mT	2	2	3	7
EMF 5 mT	2	2	3	7
EMF 8 mT	2	2	3	7

Three categories were evaluated: **A** intensity of safranin-O staining, **B** distance between cells/amount of matrix that was accumulated and **C** cell morphologies represented

Category A: Intensity of safranin-O staining. Each sample was observed with a 10× objective.

Category B: Distance between cells and amount of matrix produced by chondrocytes were assessed with a 20× objective.

Category C: Cell morphology was examined using a 40× objective. A rounded morphology was expected for chondrocytes and the presence of pyknotic or fibroblast morphology was scored as poor cell quality.

### RNA isolation

Cell pellets were disrupted under frozen conditions with 3000 rpm for 1 min, using a Micro-Dismembrator S (Sartorius, Göttingen, Germany). Total RNA was isolated directly from freeze-milled preparations, using 1 ml QIAzol Lysis Reagent (Qiagen, Hilden, Germany). After addition of 0.2 ml chloroform (Sigma-Aldrich, Steinheim, Germany), samples were shaken and incubated at RT for 10 min. For phase separation, samples were centrifuged at 15000 g for 20 min at 4 °C and the aqueous phase was transferred to a fresh tube.

Total RNA precipitation was performed by mixing 0.5 ml Isopropanol (Sigma-Aldrich, Steinheim, Germany) with the aqueous phase. After incubation at RT for 10 min samples were centrifuged at 15000 g over night at 4 °C. RNA pellets were washed twice with 1 ml 75% Ethanol (Merck, Darmstadt, Germany) and centrifuged at 15000 g for 20 min at RT. After drying pellets, total RNA was dissolved in 32 μl RNAse free water (Gibco, Darmstadt, Germany). Concentration and purity was determined by Nanodrop (ND-1000, Thermo Fisher, Waltham, MA).

### Quantitative real-time polymerase chain reaction (qRT-PCR)

For cDNA synthesis, 0.5 μg total RNA from each pellet culture (3 with EMF treatment and 3 without treatment for each patient) was reverse-transcribed using the QuantiTect Reverse Transcription Kit (Quiagen, Hilden, Germany). QRT-PCR was performed using a LightCycler 96 System (Roche Diagnostics, Mannheim, Germany). Each reaction contained 5 μL of FastStart Essential DNA Green Master Mix (Roche Diagnostics, Mannheim, Germany), 2.5 μl of 1:3 diluted cDNA and 0.3 μl (300 nM), for test genes or 0.5 μl (500 nM), for reference gens of primer in a 10 μl final volume.

The following primers were used: Glycerinaldehyd-3-phosphat-dehydrogenase (*GAPDH*) [19], collagen type II α_1_ chain (*COL2A1*) [20], collagen type 1 α_1_ chain (*COL1A1*) [21], cartilage-specific proteoglycan core protein (*ACAN*) [20] and SRY (sex determining region Y)-box 9 (*SOX9*) [20] (Table 2).

**Table 2:** Gene sequences of primer pairs and annealing temperatures

Gene	Sequence	Annealing temperature
GAPDH forward	TGC ACC ACC AAC TGC TTA GC	60 °C
GAPDH reverse	GGC ATG GAC TGT GGT CAT GAG	60 °C
COL2A1 forward	GTT ATC GAG TAC CGG TCA CAG AAG	65 °C
COL2A1 reverse	AGT ACT TGG GTC CTT TGG GTT TG	65 °C
ACAN forward	CAG CAC CAG CAT CCC AGA	65 °C
ACAN reverse	CAG CAG TTG ATT CTG ATT CAC G	65 °C
COL1A1 forward	TGA CCT CAA GAT GTG CCA CT	65 °C
COL1A1 reverse	ACC AGA CAT GCC TCT TGT CC	65 °C
SOX9 forward	AGA CCT TTG GGC TGC CTT AT	60 °C
SOX9 reverse	TAG CCT CCC TCA CTC CAA GA	60 °C

**Fig. 3:**
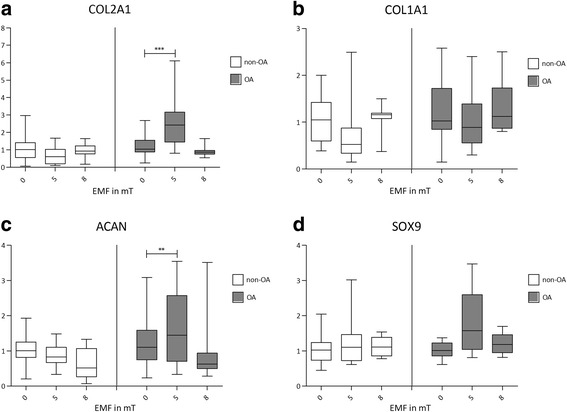
Alteration of normalized gene expression of COL2A1 (**a**), COL1A1 (**b**), ACAN (**c**) and SOX9 (**d**) to GADPH expressed by pellets of OA (*n* = 12 with 5 mT and *n* = 9 with 8 mT) and non-OA patients (*n* = 9 with 5 mT and 8 mT) after 7 days in a 5 mT or 8 mT EMF normalized to the respective control group without (0 mT) EMF exposition

Thermal cycling parameters included a denaturation step for 10 min,40 cycles of 95 °C for 10 s, 60 °C /65 °C for 10 s and 72 °C for 15 s. Reactions for each pellet were performed in triplicate and the mean of relative quantification values was calculated by using the delta-delta Ct method [22] with *GAPDH* as the reference gene. Box-plots (Fig. 3) of cartilage specific genes and *COL1A1* were established by normalizing qRT-PCR data of pellets with 5 and 8 mT EMF treatment to the mean of control pellets without EMF treatment.

### Statistical analysis

All RNA variables were described graphically by box-and-whisker plots and numerically using appropriate measures of location and dispersion. Due to the positive skew of their distributions of our outcome variables, we used the decadic logarithms instead of the original values as dependent variables in our models. The appropriateness of the log transformation was checked graphically using the residual plots for the resulting models. The effects of diagnosis, electromagnetic field strength, and their interaction with the log-transformed outcome variables were explored by means of mixed effects model with a random intercept per patient using the MIXED procedure of the Statistical Analysis System SAS, version 9.4 for Windows (SAS Institute, Cary, NC, USA). *P* < 0.05 were regarded as statistically significant.

## Results

### Histology and immunohistochemistry

Positive staining for safranin-O and alcian blue were found in all pellet cultures. All pellet cultures showed signs of collagen type II and type I with differences in density. Representative images of pellet cultures are shown in Fig. 2 with pellet sections from one OA patient cultured without EMF (Fig. 2a, e, i, m) and one cultured with 5 mT EMF (Fig. 2b, f, j, n). Figure 2 also shows pellet sections from one non-OA patient cultured without EMF (Fig. 2c, g, k, o) and one with EMF 5 mT (Fig. 2d, h, l, p).

Comparing pellet cultures of non-OA to OA chondrocytes, non-OA cultures showed a more homogenous and compact structure than pellets from OA chondrocytes. A stronger staining for collagen type I was observed in OA cultures (Fig. 2m, n). OA cultures treated with specific 5 mT EMF showed a similar staining for collagen type II to non-OA cultures (Fig. 2j, k, l) whereas untreated OA pellets showed less staining for collagen type II (Fig. 2i).

OA pellets treated with 8 mT showed no differences in staining compared to the control OA group without EMF (data not shown).

No differences in staining intensity for collagen type II and collagen type I were found for pellet cultures of non-OA chondrocytes which had been treated with EMF (5 mT/8 mT) versus the control non-OA group (5 mT data in Fig. 2; 8 mT data not shown).

### Visual histological grading

Table 1 shows results of the visual histological grading system. Pellets from OA patients with 5 mT EMF treatments had a higher score in category A and B than in the OA control group without EMF treatment. The pellets from OA patients treated with 5 mT EMF did not reach the scores from pellets of non-OA patients. OA pellets treated with 8 mT showed the same score as the untreated controls. Pellets from non-OA patients showed no difference in all three categories (0 mT/5 mT/8 mT) and presented the best cartilage-like cell morphology with strong staining for collagen type II, formation possessed the rounded morphology typical of chondrogenic cells and are surrounded by an extracellular matrix (Table 1).

### Quantitative real-time PCR

All pellet cultures from OA patients and non-OA patients expressed the cartilage specific markers *COL2A1*, *ACAN* and *SOX9* mRNA and the unspecific *COL1A1* mRNA (Fig. 3a-d).

OA cultures from the 5 mT EMF treatment group presented with significantly higher expression (*p* < 0.001) of COL2A1 and ACAN (*p* < 0.01) than the OA control group with 0 mT EMF treatment (Fig. 3a, c). In contrast, no changes in gene expression were observed in pellets from non-OA patients.

OA cultures treated with 8 mT EMF showed also no significant differences in gene expression compared to OA control cultures with 0 mT EMF treatments (Fig. 3a-d).

No significant changes in gene expression were observed for pellets from non-OA patients treated with or without 8 mT EMF (Fig. 3a-d).

## Discussion

Effects of 5 mT and 8 mT EMF on OA and non-OA chondrocytes were examined in a 3D culture system. The 5 mT EMF increased *COL2A1* and *ACAN* gene expression of OA chondrocytes. The 8 mT EMF did not show any effects on OA and non-OA chondrocytes gene expression. The effects of EMFs on chondrocytes are inconsistent in the literature [4, 23]. A specifically beneficial EMF is difficult to characterize as in the literature electromagnetic fields with a broad range of intensity have been used in similar settings. EMFs in the range from 0.5 up to 5 mT seem to generate positive results on cartilage [11]. Within this study a 5 mT EMF, which had previously been shown to exert beneficial effects on the chondrogenic differentiation of hMSCs [12], was examined versus an EMF of 8 mT. No effects of the 8 mT EMF on gene expression was observed in OA nor in non-OA chondrocytes. It can therefore be postulated that although there is a field spectrum for beneficial effects of EMF on cartilaginous cells, it is possible to reduce EMF effects by slight adjustments to electromagnetic field.

As EMF effects depend very much on multiple parameters including frequency, magnetic field peak amplitude as well as exposure length [24] we were interested whether EMF effects also vary with on cell quality. To our knowledge, studies comparing EMF effects on human OA to non-OA cartilage are lacking. In previous published studies EMFs were applied to OA cartilage explants, where they were shown to increase proteoglycan synthesis after 7 days [7]. EMF effects were also examined in bovine articular cartilage explants, where an EMF (2.3 mT, 60 Hz), increased proteoglycan synthesis both under basal conditions and in the presence of interleukin-1 [24]. Positive effects of 1, 2 and 3 mT 60 Hz EMFs on the expression of cartilage specific genes and glycosaminoglycan synthesis were also observed in non-OA bovine chondrocytes [8]. When particular parameters were used, EMFs were even able to prevent degenerative changes in cartilage explants with a potency almost reaching low-intensity pulsed ultrasound research [25].

During this study EMF effects on human OA and non-OA chondrocytes were cleary divergent. The application of 5 mT EMF resulted in an upregulation of *COL2A1* and *ACAN* gene expression in OA chondrocytes. In contrast to OA chondrocytes, non-OA chondrocytes did not seem to significantly profit from EMF.

Previoussimilar results were shown in a study of hMSCs where EMF effects were only observed under suboptimal cell culture conditions and EMF effects were dependent on the cellular quality of the hMSCs [12]. Why EMF stimulation is mainly effective in degenerated or suboptimal chondrogenic cells remains to be answered. Variable reasons are possible to explain the stronger influence of EMF on degenerated chondrocytes.

Regarding the pathogenesis of OA adenosine receptors (ARs), A_2A_ and A_3_ play an important role in the anti-inflammatory pathway by decreasing expression of nuclear factor κB (NF-κB) a transcriptional factor of inflammatory cytokines such as interleukin-1 beta (IL-1ß), interleukin-6 (IL-6) and tumornecrosis factor alpha (TNF-α) [26]. EMFs have been described to increase anti-inflammatory effects by inducing up-regulation of A_2A_ and A_3_ ARs [27, 28].

An other mechanism for a re-improvement of chondrogenic potential through EMF may be increased growth factor synthesis [29]. EMFs act on transforming growth factor beta 1 (TGF-β_1_) -growth factor release and may thereby restore the chondrogenic potential of degenerated or suboptimal chondrogenic cells. In addition, EMFs may have anti-apoptotic effects by decreasing the expression of proteins involved in the pro-apoptotic pathway and simultaneously increasing expression of anti-apoptotic proteins [30]. EMF treatment seems to improve suboptimal or degenerate cellular conditions for chondrocytes [31, 32]. In the absence of chondrocyte stress EMFs may be ineffective which may in part explain the lack of EMF effect in some studies. It also needs to be evaluated whether degeneration of tissue or the age of the donor are responsible for the EMF effects. For general statements about degeneration and EMF effects, further studies are necessary to clearly define this mechanism.

## Conclusions

The application of 5 mT EMFs has a positive effect on degenerated OA chondrocytes by increasing expression of *COL2A1* and *ACAN*. The effect of 5 mT EMFs on non-OA chondrocytes is markedly less pronounced. 8 mT EMF did not exert an effect on OA or on non-OA chondrocytes. Further studies are necessary to evaluate EMF effects in different settings, which may be a valuable tool in treating OA patients.

## Abbreviations

ABC: Avidin-biotin-peroxidase-complex
ACAN: Cartilage-specific proteoglycan core protein
ARs: Adenosine receptors
COL1A1: Collagen type 1 alpha_1_ chain
COL2A1: Collagen type II alpha_1_ chain
DAB: 3.3 Diamino benzidine tetrahydrochlorhide
DMEM: Dulbecco’s modified eagle’s medium
EMFs: Electromagnetic fields
GAPDH: Glycerinaldehyd-3-phosphat-dehydrogenase
hMSCs: Human mesenchymal stem cells
IHC: Immunohistochemistry
IL--1β: Interleukin-1 beta
IL-6: Interleukin-6
mABs: Monoclonal mouse antibodies
NF- κB: Nuclear factor κB
OA: Osteoarthritis
PBS: Phosphate buffered saline
qRT-PCR: Quantitative real-time polymerase chain reaction
RT: Room temperature
SOX9: SRY (sex determining region Y)-box 9
TGF-β1: Transforming growth factor beta 1
TNF-α: Tumornecrosis factor alpha

## References

[1] Conaghan PG, Kloppenburg M, Schett G, Bijlsma JWJ, Comm EOAH (2014). Osteoarthritis research priorities: a report from a EULAR ad hoc expert committee. Ann Rheum Dis.

[2] Li S, Yu B, Zhou D, He C, Zhuo Q, Hulme JM. Electromagnetic fields for treating osteoarthritis. Cochrane Database Syst Rev. 2013:CD003523. 10.1002/14651858.CD003523.pub2 PMC13080287 24338431

[3] Hunter DJ, Schofield D, Callander E (2014). The individual and socioeconomic impact of osteoarthritis. Nat Rev Rheumatol.

[4] Negm A, Lorbergs A, Macintyre NJ (2013). Efficacy of low frequency pulsed subsensory threshold electrical stimulation vs placebo on pain and physical function in people with knee osteoarthritis: systematic review with meta-analysis. Osteoarthr Cartil.

[5] De Mattei M, Pasello M, Pellati A, Stabellini G, Massari L, Gemmati D, Caruso A (2003). Effects of electromagnetic fields on proteoglycan metabolism of bovine articular cartilage explants. Connect Tissue Res.

[6] Fini M, Torricelli P, Giavaresi G, Aldini NN, Cavani F, Setti S, Nicolini A, Carpi A, Giardino R (2008). Effect of pulsed electromagnetic field stimulation on knee cartilage, subchondral and epyphiseal trabecular bone of aged Dunkin Hartley guinea pigs. Biomed Pharmacother.

[7] Ongaro A, Pellati A, Masieri FF, Caruso A, Setti S, Cadossi R, Biscione R, Massari L, Fini M, De Mattei M (2011). Chondroprotective effects of pulsed electromagnetic fields on human cartilage explants. Bioelectromagnetics.

[8] Hilz FM, Ahrens P, Grad S, Stoddart MJ, Dahmani C, Wilken FL, Sauerschnig M, Niemeyer P, Zwingmann J, Burgkart R (2014). Influence of extremely low frequency, low energy electromagnetic fields and combined mechanical stimulation on chondrocytes in 3-D constructs for cartilage tissue engineering. Bioelectromagnetics.

[9] Graziana A, Ranjeva R, Teissie J (1990). External electric-fields stimulate the electrogenic calcium sodium exchange in plant-protoplasts. Biochemistry.

[10] Lee EWC, Maffulli N, Li CK, Chan KM (1997). Pulsed magnetic and electromagnetic fields in experimental Achilles tendonitis in the rat: A prospective randomized study. Arch Phys Med Rehabil.

[11] Nelson FR, Zvirbulis R, Pilla AA (2013). Non-invasive electromagnetic field therapy produces rapid and substantial pain reduction in early knee osteoarthritis: a randomized double-blind pilot study. Rheumatol Int.

[12] Mayer-Wagner S, Passberger A, Sievers B, Aigner J, Summer B, Schiergens TS, Jansson V, Muller PE (2011). Effects of low frequency electromagnetic fields on the chondrogenic differentiation of human mesenchymal stem cells. Bioelectromagnetics.

[13] Bassen H, Litovitz T, Penafiel M, Meister R (1992). ELF in vitro eposure systems for inducing uniform electric and magnetic fields in cell culutre media. Bioelectromagnetics.

[14] Kraus W, Lechner F (1972). Healing of pseudoarthrosis and spontaneous fractures with structure-forming electrodynamic potentials. Munch Med Wochenschr.

[15] Rubin CT, Donahue HJ, Rubin JE, McLeod KJ (1993). Optimization of electric field parameters for the control of bone remodeling: exploitation of an indigenous mechanism for the prevention of osteopenia. J Bone Miner Res.

[16] Zhai M, Jing D, Tong S, Wu Y, Wang P, Zeng Z, Shen G, Wang X, Xu Q, Luo E. Pulsed electromagnetic fields promote in vitro osteoblastogenesis through a Wnt/beta-catenin signaling-associated mechanism. Bioelectromagnetics. 2016; 10.1002/bem.21961 26891468

[17] Heermeier K, Spanner M, Trager J, Gradinger R, Strauss PG, Kraus W, Schmidt J (1998). Effects of extremely low frequency electromagnetic field (EMF) on collagen type I mRNA expression and extracellular matrix synthesis of human osteoblastic cells. Bioelectromagnetics.

[18] Grogan SP, Barbero A, Winkelmann V, Rieser F, Fitzsimmons JS, O'Driscoll S, Martin I, Mainil-Varlet P (2006). Visual histological grading system for the evaluation of in vitro-generated neocartilage. Tissue Eng.

[19] Vandesompele J, De Preter K, Pattyn F, Poppe B, Van Roy N, De Paepe A, Speleman F. Accurate normalization of real-time quantitative RT-PCR data by geometric averaging of multiple internal control genes. Genome Biol. 2002;3 10.1186/gb-2002-3-7-research0034 PMC126239 12184808

[20] Varas L, Ohlsson LB, Honeth G, Olsson A, Bengtsson T, Wiberg C, Bockermann R, Jarnum S, Richter J (2007). Pennington D, et al: alpha 10 integrin expression is up-regulated on fibroblast growth factor-2-treated mesenchymal stem cells with improved chondrogenic differentiation potential. Stem Cells Dev.

[21] Zhang LM, Su PQ, Xu CX, Yang JL, Yu WH, Huang DS (2010). Chondrogenic differentiation of human mesenchymal stem cells: a comparison between micromass and pellet culture systems. Biotechnol Lett.

[22] Livak KJ, Schmittgen TD (2001). Analysis of relative gene expression data using real-time quantitative PCR and the 2(T) (−Delta Delta C) method. Methods.

[23] Li S, Yu B, Zhou D, He C, Zhuo Q, Hulme JM (2013). Electromagnetic fields for treating osteoarthritis. Cochrane Database Syst Rev.

[24] De Mattei M, Fini M, Setti S, Ongaro A, Gemmati D, Stabellini G, Pellati A, Caruso A (2007). Proteoglycan synthesis in bovine articular cartilage explants exposed to different low-frequency low-energy pulsed electromagnetic fields. Osteoarthr Cartil.

[25] Tan L, Ren Y, van Kooten TG, Grijpma DW, Kuijer R (2015). Low-intensity pulsed ultrasound (LIPUS) and pulsed electromagnetic field (PEMF) treatments affect degeneration of cultured articular cartilage explants. Int Orthop.

[26] Varani K, Padovan M, Vincenzi F, Targa M, Trotta F, Govoni M, Borea PA (2011). A2A and A3 adenosine receptor expression in rheumatoid arthritis: upregulation, inverse correlation with disease activity score and suppression of inflammatory cytokine and metalloproteinase release. Arthritis Res Ther.

[27] Ongaro A, Varani K, Masieri FF, Pellati A, Massari L, Cadossi R, Vincenzi F, Borea PA, Fini M, Caruso A, De Mattei M (2012). Electromagnetic fields (EMFs) and adenosine receptors modulate prostaglandin E(2) and cytokine release in human osteoarthritic synovial fibroblasts. J Cell Physiol.

[28] Vincenzi F, Targa M, Corciulo C, Gessi S, Merighi S, Setti S, Cadossi R, Goldring MB, Borea PA, Varani K (2013). Pulsed electromagnetic fields increased the anti-inflammatory effect of A(2)A and A(3) adenosine receptors in human T/C-28a2 chondrocytes and hFOB 1.19 osteoblasts. PLoS One.

[29] Aaron RK, Boyan BD, Ciombor DM, Schwartz Z, Simon BJ. Stimulation of growth factor synthesis by electric and electromagnetic fields. Clin Orthop Relat Res. 2004:30–7. 10.1097/00003086-200402000-00006 15021128

[30] Podda MV, Leone L, Barbati SA, Mastrodonato A, Li Puma DD, Piacentini R, Grassi C (2014). Extremely low-frequency electromagnetic fields enhance the survival of newborn neurons in the mouse hippocampus. Eur J Neurosci.

[31] Corallo C, Volpi N, Franci D, Vannoni D, Leoncini R, Landi G, Guarna M, Montella A, Albanese A, Battisti E (2013). Human osteoarthritic chondrocytes exposed to extremely low-frequency electromagnetic fields (ELF) and therapeutic application of musically modulated electromagnetic fields (TAMMEF) systems: a comparative study. Rheumatol Int.

[32] Corallo C, Battisti E, Albanese A, Vannoni D, Leoncini R, Landi G, Gagliardi A, Landi C, Carta S, Nuti R, Giordano N (2014). Proteomics of human primary osteoarthritic chondrocytes exposed to extremely low-frequency electromagnetic fields (ELF EMFs) and to therapeutic application of musically modulated electromagnetic fields (TAMMEF). Electromagn Biol Med.

